# The Physiological Action of Tobacco

**Published:** 1886-02

**Authors:** 


### The Physiological Action of Tobacco.

In the Fiske Fund Prize Dissertation, No. XXXIV., entitled "The Physiological and Pathological Effects of the Use of Tobacco," recently published by Messrs. P. Blakiston, Son & Co., of Philadelphia, the author, Dr. Hobart Amory Hare, presents the facts and arguments that have led him to the following conclusions:

1. Tobacco-smoking does not decrease the urine eliminated, but rather increases it. 2. Tobacco does retard tissue waste. 3. Tobacco and its alkaloid cause convulsions in the primary stage of the poisoning, by depressing the reflex inhibitory centers in the cord. 4. It causes the palsy of the second stage, by paralyzing (*a*) the motor nerve-trunks, (*b*) the motor tract of the spinal cord. 5. The sensory nerves are not affected by the drug. 6. Nicotine contracts the pupil, by stimulating the oculo-motor and paralyzing the sympathetic, this action being peripheral. 7. Nicotine primarily lowers the blood-pressure and pulse-rate; (*a*) secondarily, increases pressure and rate; (*b*) thirdly, decreases pressure. 8. The primary lowering of pressure and rate is due to pneumogastric stimulation, associated with vaso-motor dilatation. 9. The secondary stage is due to vaso-motor constriction and pneumogastric palsy. 10. The third stage is due to vaso-motor dilatation returning. 11. Death in poisoning from this drug is due to failure of respiration, the action of the drug being centric. 12. The blood-corpuscles are broken up and crenated by the action of the poison. 13. In death from nicotine-poisoning, the blood shows changes in *spectra*. 14. Death can be brought about by the cutaneous absorption of nicotine. 15. Tobacco increases intestinal peristalsis in moderate amounts, and produces tetanoid intestinal spasms in poisonous doses. 16. The liver seems to destroy the poison, although this destruction is participated in by any set of capillaries in other parts of the body. 17. Tobacco-smoking increases the pulse-rate, and decreases arterial pressure.—*Therapeutic Gazette.*

